# Supplementary material for: Understanding text-based persuasion and support tactics of concerned significant others
Source: PeerJ. 2015 Aug 18;3:e1151. doi: 10.7717/peerj.1151 (PMC4548473; doi:10.7717/peerj.1151)
Supplement: Appendix S1 [file peerj-03-1151-s002.pdf]

Appendices

Appendix A. MTurk Survey

Thank you for your interest in the survey. The purpose of this study is to increase our understanding of the goals people have in life and how to support individuals attempting to change a behavior.

You will complete one short survey that takes approximately 5 minutes. Your responses will be collected anonymously using Survey Monkey, an online survey hosting site.

You do not have to participate - your participation is voluntary and completely anonymous. Completing this survey involves very little risk to you. You may feel uncomfortable answering some questions. You can refuse to answer any question that you do not feel comfortable answering, and you can refuse to complete the survey by stopping at any time. If you stop the survey or skip more than three questions, you will not receive the compensation for your participation. Once you have completed your survey, you will be given a compensation code that you can copy and paste into MTurk. You will receive 50 cents for completing the survey.

IF YOU HAVE COMPLETED THIS OR ANY OF OUR PAST SURVEYS PREVIOUSLY, PLEASE DO NOT ATTEMPT TO COMPLETE THIS SURVEY. WE HAVE YOUR MTURK ACCOUNT INFORMATION AND WILL CHECK TO SEE IF WE HAVE COMPENSATED YOU IN THE PAST. IF SO, YOUR WORK ON THIS HIT WILL BE REJECTED AND YOU WILL NOT RECEIVE PAYMENT.

If you have any questions or concerns, you may contact the Principal Investigator, Dr. Fred Muench, at (212) 974-0547, or the Administrative Director of the New York State Psychiatric Institute Institutional Review Board at (212) 543-5758.

☐ Agree

☐ Disagree

For this exercise, please think of someone you are very close to and care deeply about, who you think should change a behavior in order to improve his/her life. This person could be a family member, a close friend, etc.

What is this person's relationship to you?

- ☐ Spouse/Partner
- ☐ Mother
- ☐ Father
- ☐ Sister
- ☐ Brother
- ☐ Son
- ☐ Daughter
- ☐ Grandmother
- ☐ Grandfather
- ☐ Close friend
- ☐ Coworker
- ☐ Boss

Other (please specify)

What is the personal goal you want this person to achieve? This can be anything from exercising to being more assertive to cutting down on drinking. There are no right types of goals.

What brief message might you send to this person to help them meet their goal?

What brief message might you send to this person if you knew that (s)he wasn't meeting his/her goal?

What brief message might you send to this person if you knew that (s)he had given up entirely on trying to meet his/her goal?

On a scale of 1 to 10, how would you rate your relationship with this person?

1 = Our relationship is extremely rocky (eg, we're estranged, we can't see each other without having a conflict).
10 = Our relationship is extremely close (eg, we never argue and are always there for each other)

○ 1 ○ 2 ○ 3 ○ 4 ○ 5 ○ 6 ○ 7 ○ 8 ○ 9 ○ 10

On a scale of 1 to 10, how generally motivated is this person to meet his/her goal?

1 = Not at all motivated – (s)he won't even admit it's a problem
10 = Extremely motivated – this is his/her #1 priority

○ 1 ○ 2 ○ 3 ○ 4 ○ 5 ○ 6 ○ 7 ○ 8 ○ 9 ○ 10

If you were to offer this person your support or suggestions to help them meet their goal, how do you think that they would react to you?

Select all that apply:

- ☐ (S)he would be appreciative/grateful
- ☐ (S)he would be enthusiastic/excited
- ☐ (S)he would not react to my support or suggestions at all
- ☐ (S)he would be withdrawn/detached
- ☐ (S)he would be embarrassed
- ☐ (S)he would be annoyed/irritated
- ☐ (S)he would be anxious/panicky
- ☐ (S)he would be hurt/upset
- ☐ (S)he would be angry/aggressive

Other (please specify)

If you were to offer this person your support or suggestions to help them meet their goal, do you think they would take your advice?

1 = (S)he would probably retaliate by doing the opposite of my suggestion.
10 = (S)he would probably make a huge effort to use my advice to meet his/her goal.

○ 1 ○ 2 ○ 3 ○ 4 ○ 5 ○ 6 ○ 7 ○ 8 ○ 9 ○ 10

If the 2 questions above did not seem to apply to your specific situation, please expand on how you think this person would react to you showing your support in his/her efforts to meet this goal:

On a scale of 1 to 10, how severe would the consequences be if this person doesn't reach his/her goal?

1 = (S)he would suffer no consequences whatsoever
10 = The consequences would be extremely severe (e.g. distressing long-term health or emotional consequences)

○ 1 ○ 2 ○ 3 ○ 4 ○ 5 ○ 6 ○ 7 ○ 8 ○ 9 ○ 10

On a scale of 1 to 10, how frustrated do you get with this person over the behavior you want him/her to change?

1 = I never get frustrated with him/her
10 = I get extremely frustrated with him/her

○ 1 ○ 2 ○ 3 ○ 4 ○ 5 ○ 6 ○ 7 ○ 8 ○ 9 ○ 10

How old are you?

- ☐ Under 18
- ☐ 18-21
- ☐ 22-25
- ☐ 26-30
- ☐ 31-40
- ☐ 41-50
- ☐ 51-60
- ☐ 61-70
- ☐ Over 70

Gender:

- ☐ Male
- ☐ Female

What race do you most closely identify with?

- ☐ American Indian or Alaska Native
- ☐ Asian
- ☐ Black or African American
- ☐ Native Hawaiian or other Pacific Islander
- ☐ White

Other (please specify)
